# Effects of Different Scleral Photo-Crosslinking Modalities on Scleral Stiffness and Hydration

**DOI:** 10.1167/iovs.65.8.8

**Published:** 2024-07-03

**Authors:** Lupe Villegas, James A. Germann, Susana Marcos

**Affiliations:** 1 Instituto de Óptica “Daza de Valdés,” Consejo Superior de Investigaciones Científicas, Madrid, Spain; 2 Universidad Politécnica de Madrid, Madrid, Spain; 3 The Center for Visual Science, The Institute of Optics; Flaum Eye Institute, University of Rochester, Rochester, New York, United States

**Keywords:** sclera, cross-linking, biomechanics, young's modulus (YM), swelling

## Abstract

**Purpose:**

The purpose of this study was to evaluate the biomechanical and hydration differences in scleral tissue after two modalities of collagen cross-linking.

**Methods:**

Scleral tissue from 40 adult white rabbit eyes was crosslinked by application of 0.1% Rose Bengal solution followed by 80 J/cm^2^ green light irradiation (RGX) or by application of 0.1% riboflavin solution followed by 5.4 J/cm^2^ ultraviolet A irradiation (UVX). Posterior scleral strips were excised from treated and untreated sclera for tensile and hydration-tensile tests. For tensile tests, the strips were subjected to uniaxial extension after excision. For hydration-tensile tests, the strips were dehydrated, rehydrated, and then tested. Young's modulus at 8% strain and swelling rate were estimated. ANOVAs were used to test treated-induced differences in scleral biomechanical and hydration properties.

**Results:**

Photo-crosslinked sclera tissue was stiffer (Young's modulus at 8% strain: 10.7 ± 4.5 MPa, on average across treatments) than untreated scleral tissue (7.1 ± 4.0 MPa). Scleral stiffness increased 132% after RGX and 90% after UVX compared to untreated sclera. Scleral swelling rate was reduced by 11% after RGX and by 13% after UVX. The stiffness of the treated sclera was also associated with the tissue hydration level. The lower the swelling, the higher the Young's modulus of RGX (−3.8% swelling/MPa) and UVX (−3.5% swelling/MPa) treated sclera.

**Conclusions:**

Cross-linking with RGX and UVX impacted the stiffness and hydration of rabbit posterior sclera. The Rose Bengal with green light irradiation may be an alternative method to determine the efficacy and suitability of inducing scleral tissue stiffening in the treatment of myopia.

Biomechanical and structural changes in the posterior sclera are associated with abnormal elongation of the eye, which results in myopia. Specifically, the defocus-induced retinal signaling cascade may lead to scleral remodeling, making posterior sclera more susceptible to axial elongation.^1^^,^^2^ In recent years, scleral collagen cross-linking (SXL) has been proposed as a therapeutic treatment for the prevention of progressive myopia using photo-crosslinking^3^^–^^5^ and chemical-crosslinking.^6^^,^^7^ By taking advantage of the tissue targeting of cross-linking,^8^ it is possible to treat the equatorial sclera.^9^ If the photosensitizer and the irradiated light can reach the posterior sclera (e.g. using flexible optical waveguides^10^ or LED probes^11^), photo-crosslinking may be a viable technique to strengthen the sclera and arrest myopia.

Photo-crosslinking has been used for decades to stiffen corneal tissue^12^ and more recently to stiffen scleral tissue.^3^^,^^13^^–^^16^ In vivo SXL using a method similar to the Dresden protocol in the cornea, that is, instillation of 0.1% riboflavin and UVA light irradiation (UVX), has been shown to increase Young's modulus (YM) in rabbit^3^^,^^5^^,^^17^^,^^18^ and guinea pig^4^ sclerae. However, despite the promise of scleral stiffening, translating these methods to human trials remains challenging. Limitations include moderate inflammatory response in the treated area,^17^ the challenge of treating the posterior sclera without surgery,^8^ and retinal toxicity of the treatment light.^19^

As an alternative to UVX, Rose Bengal combined with 532-nm green light (RGX) has been shown to increase corneal stiffness, measured by tensile test^20^^–^^22^ and air-puff deformation imaging,^23^^,^^24^ and to maintain collagen fiber organization, measured by second harmonic generation microscopy.^25^ In addition, the safety of RGX have been evaluated in the cornea, showing no evidence of damage in the mid-depth cornea, retina, and iris of rabbit eyes following RGX at total irradiation energies of 68 J/cm^2^,^26^ 100 J/cm^2^,^27^^,^^28^ and 150 J/cm^2^.^21^ The results suggest that RGX is an effective and safe procedure in collagen-rich tissues and could therefore be used for scleral stiffening.

An important parameter to consider when measuring the mechanical behavior of collagenous tissue is swelling. Previous studies have investigated the effects of hydration and cross-linking on the ocular tissue stiffness using tensile tests,^29^^–^^31^ compression tests,^32^ inflation tests,^33^ and Brillouin microscopy.^34^ In UVX-crosslinked bovine^29^ and porcine^30^^,^^32^^–^^34^ corneas, a decrease in stiffness was observed with increasing hydration (based on thickness measurements). These changes in stiffness with hydration appear to be related to the glycosaminoglycan content in the ocular tissue.^35^^–^^37^

The purpose of this study was to compare the effects on the biomechanical and swelling properties of scleral tissue after photo-crosslinking by Rose Bengal with green light and riboflavin-UVA in rabbit eyes. Stress-strain curves from tensile tests were used to quantify YM of untreated and treated scleral strips (RGX and UVX). Our results showed that changes in scleral stiffness are influenced by a combination of treatment, region, and hydration.

## Materials and Methods

### Sample Preparation

**Figure 1.**
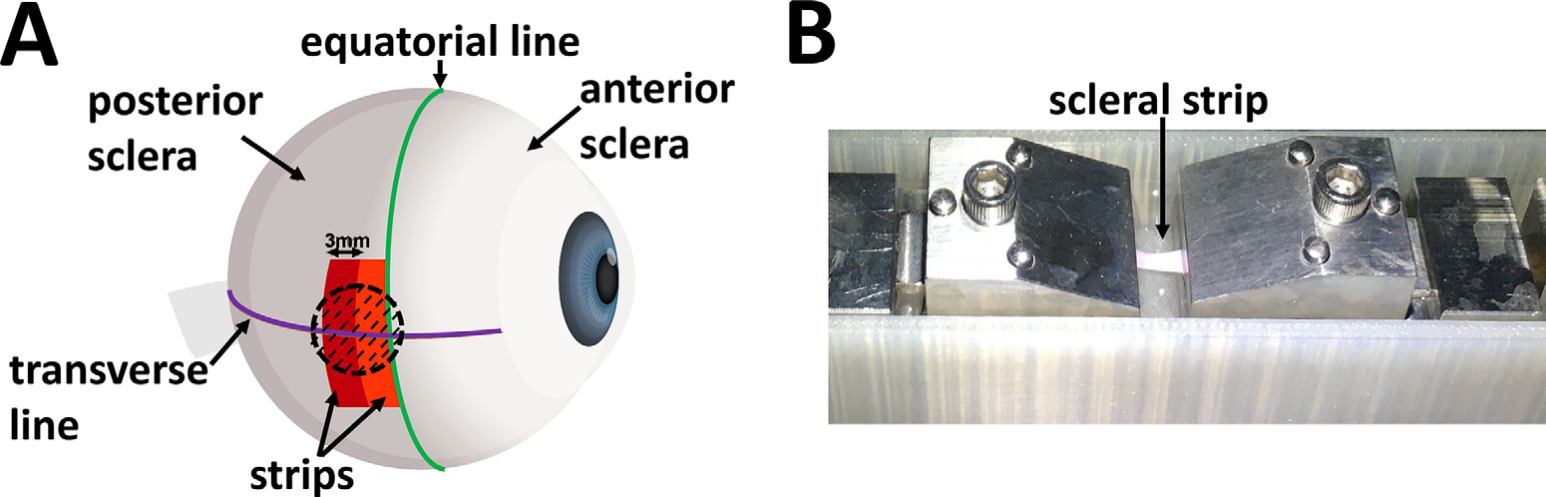
Schematic view of rabbit eyeballs showing reference lines and locations of scleral collagen cross-linking. (**A**) The equatorial line (in *green*) and the transverse line (in *purple*) were marked in all eyes to identify the anterior and posterior scleral regions. The entire globe was crosslinked on one side (nasal or temporal, *black dashed area*), the symmetric side was left as control (not shown). The *dashed circle* represents the treated area of 10-mm diameter. The figure shows the treated area with two 3-mm-wide strips. The strips were obtained from treated and untreated posterior sclera. Each strip was then mounted in a uniaxial stretcher (**B**) for the tensile test.

Forty ocular globes were obtained in pairs from adult New Zealand white rabbits (20 rabbits, 14-16 weeks old, 2-3 kg, and all females) from a farm associated with the Veterinary Faculty of the Complutense University (Madrid, Spain). The eyes were separated into groups: (1) RGX (12 eyes), (2) UVX (10 eyes), (3) hydration-RGX (10 eyes), and (4) hydration-UVX (8 eyes). Muscle and conjunctival tissue were removed. Eyes (less than 24 hours postmortem) were demarcated on to the equatorial (green line in Fig. 1A) and transverse lines (see the purple line in Fig. 1A), and placed on a custom-designed mount that exposed only the area to be treated. The treated areas (10-mm diameter) were marked on the nasal or temporal posterior sclera and cross-linked by RGX or UVX. Posterior scleral samples (3 mm × 20 mm strips) were excised from the treated and untreated areas in the equatorial direction. The retina and choroid were carefully scraped off. To reduce inter-sample variability, two strips were taken from each region, but the mean of the measurements per region was used for statistical analysis. The center of the strips was approximately 2.5 mm and 5.5 mm, respectively, from the optic nerve head toward the limbus. The thickness of all strips was carefully measured at the strip center (mean values were used) with a micrometer (digital, Mitutoyo, model 293-240-30), see Supplementary Material, Section E. The width and length were measured with a caliper (analogue, Alca, 0.05 mm, 1-150 mm). Strips were subjected to a tensile (groups 1 and 2) or hydration-tensile test (groups 3 and 4).

### Cross-Linking Techniques

In all groups, one side of the eyeball (nasal or temporal scleral region, randomly chosen) was crosslinked, and the other side was left as a contralateral control (untreated area). The globe was mounted, exposing the area to be treated. Following the initial instillation of a photosensitizer, the light source (UV lamp for UVX and green laser for RGX) irradiated only a 10 mm diameter area (see the dashed circle in Fig. 1A) on the surface of the rabbit’s eye.

RGX used 0.1% w/v of Rose Bengal (95% pure; Sigma-Aldrich, Milwaukee, WI, USA) in phosphate-buffered saline (PBS) solution (Sigma-Aldrich, Milwaukee, WI, USA) as a photosensitizer and a collimated 532-nm green laser with a 200 mW/cm^2^ irradiance (FPYL-532-200T-FC105-LED, Frankfurt Laser Company, Friedrichsdorf, Germany) for irradiation. The scleral area to be treated was immersed in Rose Bengal solution (0.1% in PBS, 0.5 mL) for 120 seconds, irradiated for 200 seconds, immersed again in Rose Bengal solution for 30 seconds, and irradiated for an additional 200 second,^20^^,^^23^ for a total treatment of less than 10 minutes. Total fluence was 80 J/cm^2^.

UVX used 0.1% w/v of riboflavin solution (Ribocross TE, IROS Srl, Napoli, Italy) as a photosensitizer and a collimated 365-nm UVA lamp at 3 mW/cm^2^ (IROC, Zurich, Switzerland) for irradiation. The scleral area was instilled with riboflavin solution every 5 minutes for 30 minutes, and then riboflavin was instilled every 5 minutes with UVA irradiation for an additional 30 minutes,^12^ for a total treatment of 60 minutes. Total fluence was 5.4 J/cm^2^.

### Mechanical Testing

#### Tensile Test

Mechanical testing was used to test scleral stiffness in two different conditions: strip location (nasal and temporal), and SXL method (RGX and UVX). Posterior scleral strips (43 from the RGX group, and 32 from the UVX group) were collected and tested immediately after SXL method (less than 30 minutes of storage in a custom humidity chamber). Strips were mounted in a uniaxial stretcher (Ustretch, CellScale, Waterloo, ON, Canada). The strips were immersed in PBS during the tests (Fig. 1B). The free length between the clamps was 6 mm before stretching. The strips were subjected to 5 load/unload cycles at a rate of 1.0 mm/min with a preload force of 0.05N during the first cycle only. The force-displacement curves indicated stable preconditioning^38^ by the last cycle, and these measurements were used to calculate stress-strain curves for all strips.

#### Hydration-Tensile Test

This method aims to evaluate the effect of dehydration/rehydration on scleral stiffness that may be induced by the tensile method itself,^29^^,^^31^ and/or by the crosslinking techniques.^29^^,^^30^ Eyes from the hydration-RGX and hydration-UVX groups were used for this test. Strips (40 from the hydration-RGX and 32 from the hydration-UVX groups) were collected from untreated and treated areas of each eye. The strips were divided into two groups according to rehydration time: 40 minutes and 100 minutes. The samples were dehydrated (during 24 hours), rehydrated by instillation of 10 µL/10 minutes PBS, and mounted on a uniaxial stretcher; for more information, see Supplementary Material, Section F. During the rehydration period, strip weights were measured every 10 minutes using a precision balance (digital, OHAUS EX124/AD, 0.1 mg). Strips were processed in a controlled environment at 21 ± 2°C and 37 ± 1% humidity (digital, CLEWARE humidity sensor, 0.01⁰C/0.01% RH) during all procedures.

### Strain and Stress Analysis

Force-displacement data were obtained from tensile tests. Tensile stress σ was calculated as the force applied to the cross-sectional area (thickness × width of the scleral strip center), and the strain ε was determined as the ratio of strip elongation to the initial length. The stress-strain curves of the loading cycles were fit by the exponential function σ  =  *A*(*e*^*B*ε^  −  1),^39^ where *A* and *B* are fitting constants obtained from a nonlinear least-squares method. Therefore, YM at a specific strain was defined by $YM\;=\;\frac{d\sigma \;}{dɛ}=AB\left(e^{Bɛ}\right)$. To easily compare variations in scleral stiffness, YM was calculated at 8% strain (see Supplementary Material, Section A).

### Calculation of Swelling Ratio and Swelling Rate

The effects of dehydration/rehydration were quantified by the swelling ratio, defined as the percentage of PBS uptake due to the increase in tissue weight. The swelling ratio was determined by the equation $\text{swelling}\;\text{ratio}\left(\%\right)=\frac{W_{wet}-W_{dry}}{W_{dry}}\;x\;100$, in which the weights of scleral strips before *W_dry_* (dehydrated) and after *W_wet_* (rehydrated) soaking in PBS were compared. Subsequently, the *swelling* *rate* (%/*min*) was estimated as the slope of the linear part of the swelling-time curves. The swelling rate quantified the speed of weight increase of the scleral strip.

### Statistical Analysis

The data were analyzed using IBM SPSS Statistics (version 29.0). Repeated-measures ANOVAs were performed to evaluate differences in YM and in swelling rate in the respective groups. One-way ANOVA was performed to evaluate the difference between treatments (RGX versus UVX). Multiple comparisons were adjusted by Bonferroni corrections. Means and standard deviations of outcome variables were calculated for treated and untreated groups. Significance was set at a *P* value of 0.05.

## Results

### Increase in Stiffness after RGX and UVX Methods

**Figure 2.**
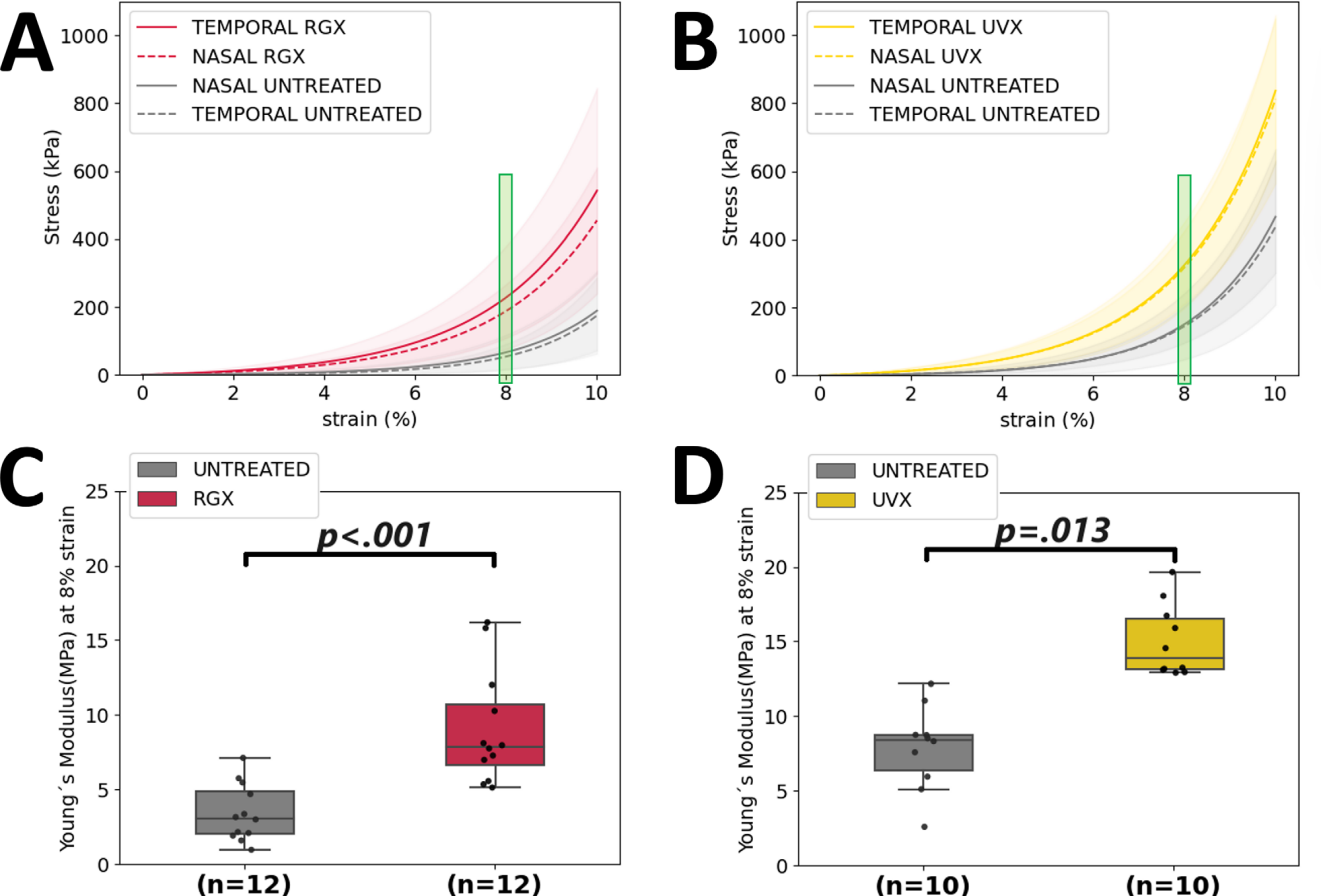
Increase in posterior scleral stiffness after RGX and UVX treatment. *Above*: Uniaxial stress-strain results for untreated and crosslinked scleral strips. Scleral strips were obtained from the posterior temporal and nasal regions. (**A**) RGX treated scleral strips (*red curves*) and (**B**) UVX treated strips (*yellow curves*) and their respective contralateral untreated samples (*gray curves*). Young's modulus was calculated from the slope of the stress-strain curves at 8% strain (*green bar*). *Below*: Estimated Young's modulus for untreated (*gray boxes*) and treated scleral strips represented as boxplots, RGX-treated eyes (*red boxes*, *n* = 12 eyes) (**C**) and UVX-treated eyes (*yellow boxes*, *n* = 10 eyes) (**D**). The *P* values are shown in the graph for significant differences. Data points are shown as *black circles*. Each data point is the average stiffness of the two strips taken per region in each eye.

Scleral stiffness increased after RGX and UVX treatment compared to untreated sclera. Tensile strength (Figs. 2A, 2B) at 8% strain was 247% (209 ± 124 kPa vs. 60 ± 46 kPa) higher in RGX-treated, and 117% (321 ± 117 kPa vs. 148 ± 78 kPa) higher in UVX-treated eyes. This resulted in a statistically significant (*P* ≤ 0.013) increase in YM (Figs. 2C, 2D) after RGX (9.5 ± 4.5 MPa) and UVX (15.0 ± 2.4 MPa) in comparison to untreated sclera (4.1 ± 2.2 MPa and 7.9 ± 2.8 MPa, respectively) from the same eye. There were no statistically significant differences in YM between temporal and nasal regions in untreated sclera (6.3 ± 1.4 MPa and 5.3 ± 1.6 MPa, *P* < 0.991, *n* = 11), or post-RGX (9.6 ± 2.1 MPa and 9.3 ± 1.7 MPa, *P* < 0.906, *n* = 6), or post-UVX (15.2 ± 1.2 MPa and 14.8 ± 1.1 MPa, *P* < 0.423, *n* = 5; see Supplementary Table B1).

### Influence of the Treatment in the Hydration Process and Stiffness

**Figure 3.**
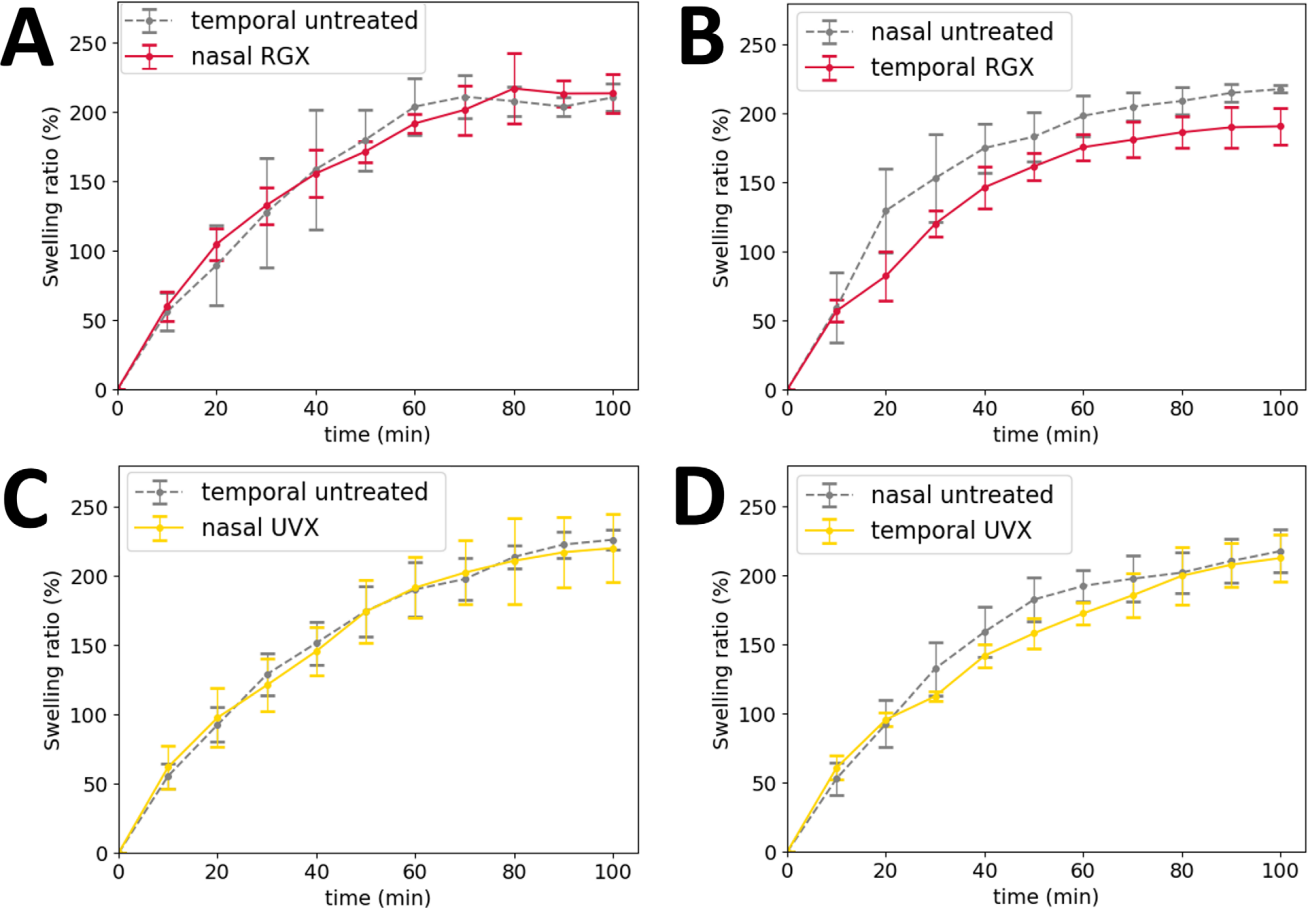
Swelling ratio in function of time of scleral strips from treated and untreated scleral tissues. (**A**) and (**B**) Swelling-time curves for RGX-treated (*red curves*) compared to contralateral untreated (*gray curves*) tissues in nasal and temporal regions, respectively. (**C**) and (**D**) Swelling-time curves for UVX-treated (*yellow curves*) compared to corresponding contralateral untreated (*gray curves*) tissues in nasal and temporal regions, respectively. There were no clear differences between swelling-time curves in any of the cases, except for the temporal region treated with RGX (**B** in the figure), where the swelling ratio decreased by 8% at 100 minutes of rehydration.

**Figure 4.**
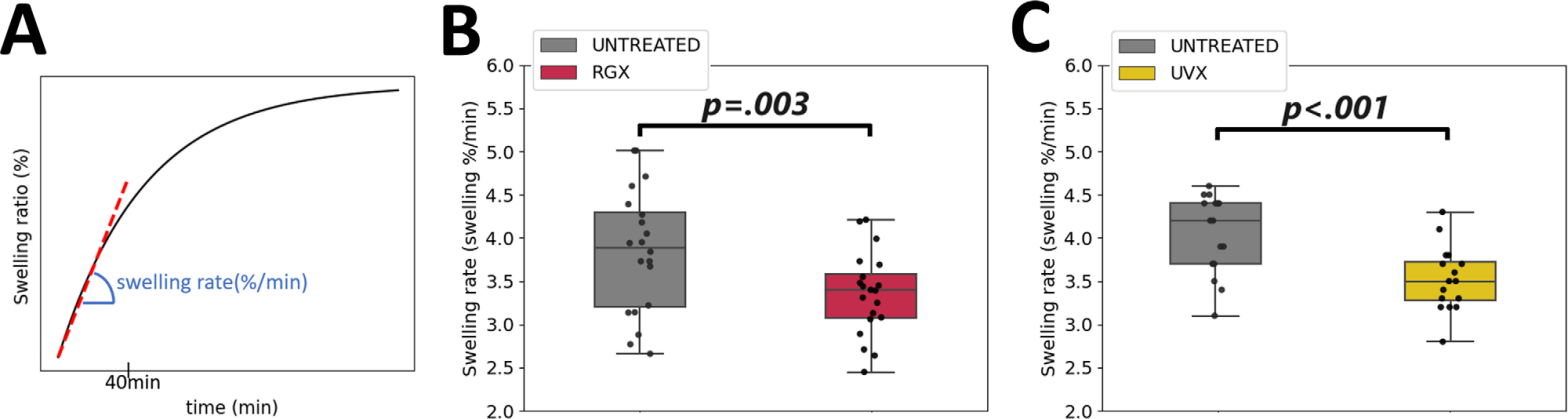
Reduction of swelling rate in RGX and UVX crosslinked scleral tissues. (**A**) Representation of the shape of a swelling-time curve after controlled PBS rehydration of scleral strips. Swelling rate was calculated from the slope of the swelling-time curves for each scleral strip in units of percentage saline uptake per minute. (**B**) Shown are boxplots of the swelling rate of untreated (*gray box*, 20 strips) and RGX-treated (*red box*, 20 strips) scleral tissues. (**C**) Boxplots show the swelling rate of untreated (*gray box*, 16 strips) and UVX-treated (*yellow box*, 16 strips) scleral tissues. Data points for each strip are shown as *black circles*. The *P* values are shown for significant differences.

The swelling ratio was estimated to investigate differences in the PBS uptake in the treated (RGX or UVX) sclera compared to the untreated sclera. The swelling curves (Fig. 3) showed an approximately linear behavior up to 40 minutes of rehydration and a constant value at 100 minutes of rehydration in the untreated tissue (217.6 ± 13.6%) and in the treated tissue (205.9 ± 23.5%). There were no clear differences in the swelling-time curves between RGX-treated (see Figs. 3A, 3B) or UVX-treated (see Figs. 3C, 3D), and untreated tissues. However, it was possible to estimate variations after examining the initial linear behavior of the curves using the swelling rate (see Fig. 4A). Untreated sclera absorbed saline at least 12% faster than treated tissue (Figs. 4B, 4C). In particular, the swelling rate was significantly higher (*P* ≤ 0.003) in untreated sclera (3.8 ± 0.7%/min and 4.0 ± 0.5%/min) compared to RGX-treated (3.4 ± 0.5%/min) and UVX-treated (3.5 ± 0.4%/min) sclera, see Supplementary Table D1. These results suggest that the RGX and UVX modified the swelling properties of the scleral tissue by reducing its swelling rate.

**Figure 5.**
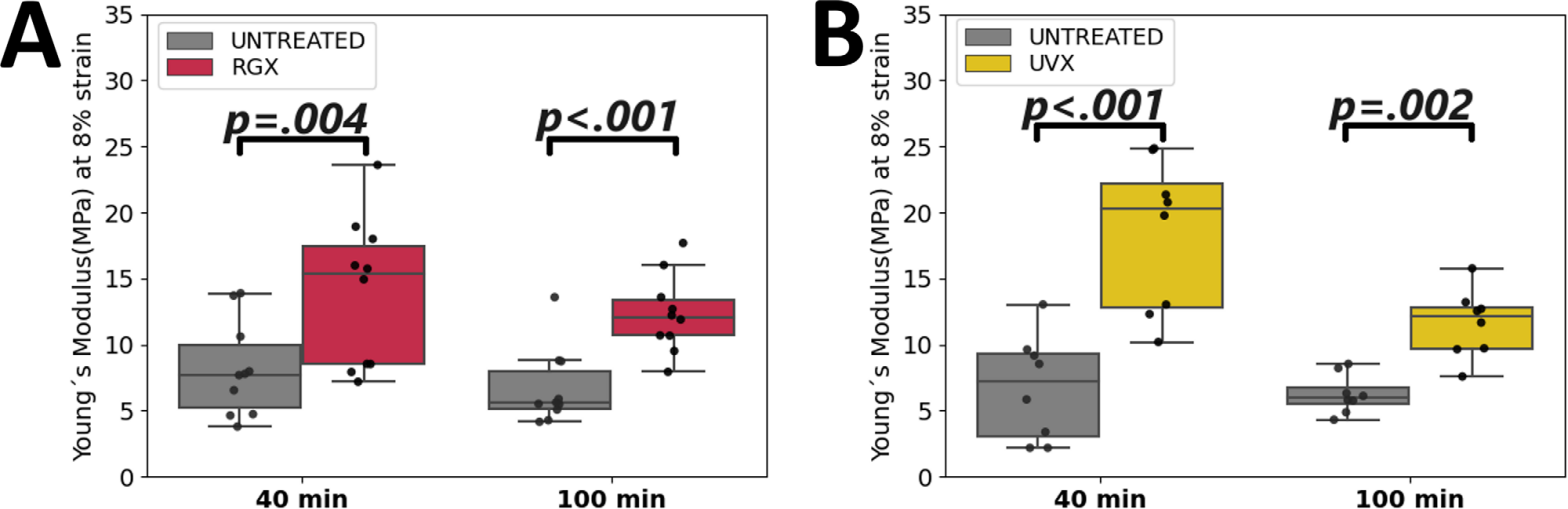
Loss of stiffness after prolonged rehydration of scleral tissue. Boxplots show Young's modulus at 8% strain of posterior sclera (**A**) for RGX-treated (*red box*, 10 strips per group) and contralateral untreated (*gray box*, 10 strips per group) tissues, and (**B**) for UVX-treated (*yellow box*, 8 strips per group) and their respective untreated (*gray box*, 8 strips per group) tissues. Strips were first rehydrated for 40 or for 100 minutes, and then tensile tested. The *P* values for significant differences between the respective groups are shown in the graph. *Black dots* indicate data points, each representing a different strip.

To determine whether scleral stiffness was also affected after rehydration, 40-minute and 100-minute rehydrated strips were subjected to tensile tests. In the overall comparison, up to at least 2-fold higher stiffness (*P* ≤ 0.004) was observed after SXL (Figs. 5A, 5B), see Supplementary Table B2. In the 40-minute rehydrated samples, YM was higher and showed a higher dispersion in RGX-treated (13.9 ± 5.6 MPa) and UVX-treated (18.4 ± 5.7 MPa) sclera. In contrast, YM was lower and showed less dispersion in both RGX (12.3 ± 2.9 MPa) and UVX (11.6 ± 2.5 MPa) 100-minute rehydrated tissues. Thus, scleral stiffness appears to be influenced by the hydration level, with longer rehydration times resulting in lower YM, although in all cases, the SXL tissue is statistically significantly stiffer than the untreated tissue.

### Swelling Ratio and Young's Modulus Correlation

**Figure 6.**
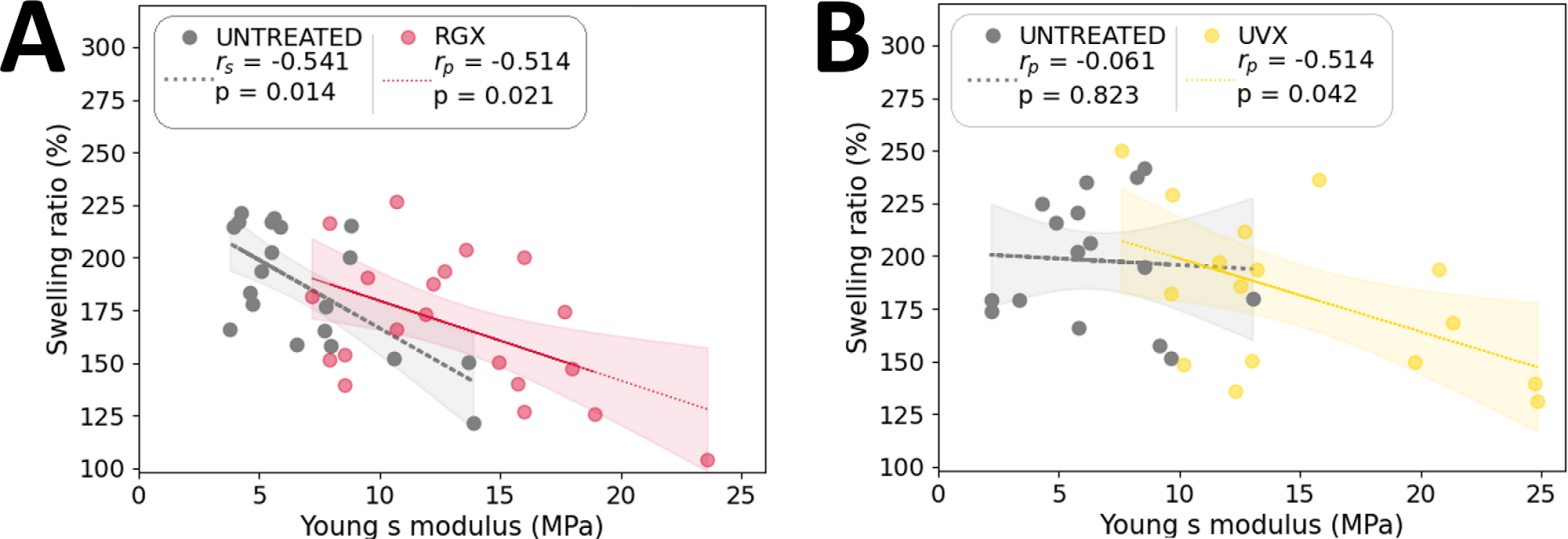
Inverse correlation between swelling and stiffness of treated and untreated sclera. Swelling ratio before stretching and the corresponding Young's modulus at 8% strain of each strip are shown as scatterplots for the untreated sclera (*gray dots*), (**A**) RGX-treated sclera (*red dots*), and (**B**) UVX-treated sclera (*yellow dots*). *Dashed lines* represent the corresponding linear regressions, and shaded areas represent 95% confidence intervals for untreated and treated data. Spearman or Pearson correlation coefficients and corresponding *P* values are shown in the *upper left corner*.

The relationship between swelling and stiffness was tested by measuring swelling before tensile test (see Fig. 3). Measurements of the final swelling ratio (see data from Fig. 3) before stretching and YM (see data from Fig. 5) were inversely correlated in RGX (*r* = −0.514, *P* = 0.021) and UVX (*r* = −0.514, *P* = 0.042) sclera, and in untreated sclera (*r* = −0.541, *P* = 0.014) of RGX-treated eyes. As expected, the lower the swelling, the higher the stiffness in both treated and untreated sclera (Fig. 6). Correlations between swelling ratio and YM were statistically significant in all cases except in the untreated sclera of UVX-treated eyes (likely due to the small sample effect).

### Comparisons Between RGX and UVX Treatments

**Figure 7.**
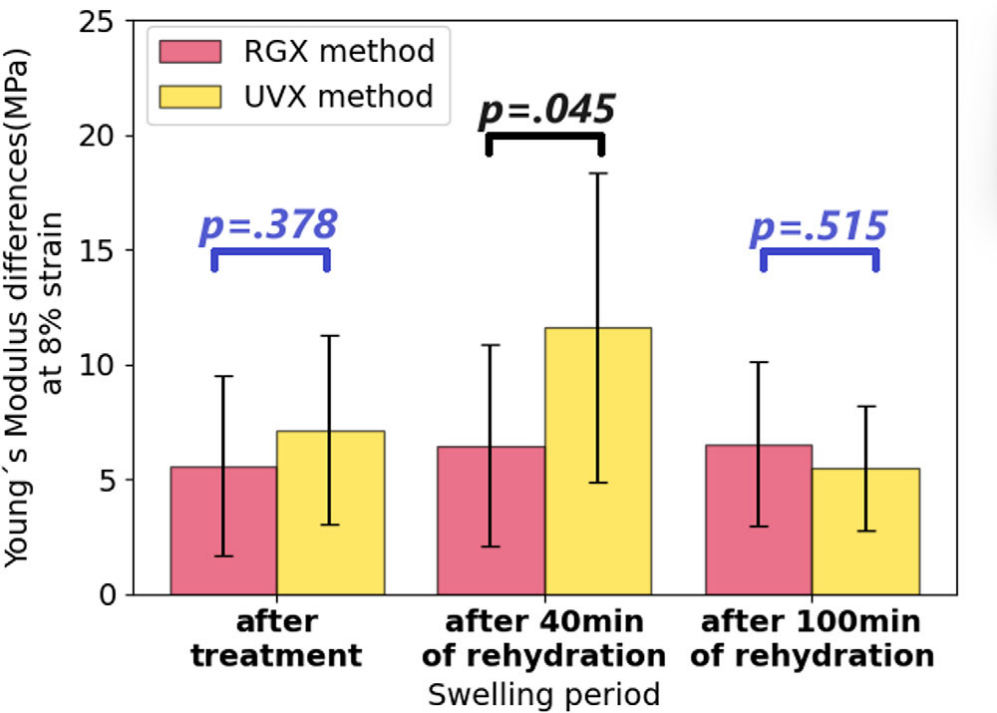
Comparison of stiffness differences. The bar plot shows the differences in Young's modulus between treated and untreated tissue for RGX-treated eyes (*red bars*) and UVX-treated eyes (*yellow bars*). Stiffness was compared for strips stretched immediately after treatment, after 40 minutes and after 100 minutes of rehydration. The *P* values are shown in *blue* (not statistically significant) and *black* (statistically significant). Values in bar plots are presented as mean ±  standard deviation.

Comparisons of YM after RGX and UVX (see Fig. 7, and data from Figs. 2 and 5) were done for all treated samples for measurements performed immediately after treatment and following 40 and 100 minutes of rehydration. Both treatments increased stiffness, with no statistically significant differences between the two, except after 40-minute rehydration (*P* = 0.045), where UVX shows higher YM values. The results suggest an interplay between hydration and material mechanical changes in the application of scleral UVX.

## Discussion

We have shown that the RGX technique effectively induces posterior scleral stiffening in rabbit tissue. Both RGX (532 nm, 80 J/cm²) and UVX (365 nm, 5.4 J/cm²) methods increased YM similarly compared to untreated sclera immediately after cross-linking and after longer (up to 100 minutes) hydration times. YM at 8% strain was 132% higher in RGX-treated sclera (9.5 ± 4.5 MPa vs. 4.1 ± 2.2 MPa) and 90% higher in UVX-treated sclera (15.0 ± 2.4 MPa vs. 7.9 ± 2.8 MPa) than in untreated sclera. In addition, RGX and UVX modified the scleral swelling properties by reducing the swelling rate by 11% and 13%, respectively. The treated scleral stiffness was also affected by hydration levels. In particular, there was an inverse correlation between the swelling ratio and YM of the treated sclera. The lower the swelling, the higher the stiffness in RGX (−3.8% swelling/MPa) and UVX (−3.5% swelling/MPa) treated sclera. Scleral tissue stiffness was influenced by a combination of crosslinking method and tissue hydration levels in specific regions of the sclera.

The 2.3-fold and 1.9-fold increases in scleral YM after RGX and UVX, respectively, are comparable to values reported after UVX of 1.4-5.6-fold^5^^,^^14^^,^^16^^,^^17^ in rabbit, 2.5-fold in porcine,^13^ and 2-fold in human sclera.^15^ Interestingly, the only prior report of scleral stiffness after Rose Bengal (and white light irradiation for 60 minutes) showed no difference from untreated tissue.^13^ An obvious difference between the previous RGX study and ours is the use of a broad-spectrum source by Wollensak et al.^13^ as opposed to a narrow bandwidth around the excitation wavelength in our study. Singh et al.^40^ also found no increase in estimated YM after RGX (560 nm, 150 J/cm^2^) in rabbit cornea using optical coherence elastography (OCE). Remarkably, OCE measures the wave propagation speed along the corneal/scleral surface, a behavior that is roughly dominated by the shear modulus,^41^ unlike uniaxial tests that provide a direct quantification of YM. In fact, by separately defining in-plane (approximately Young's modulus) and out-of-plane (approximately shear modulus) elastic moduli, the recent elastic wave propagation model,^41^ has allowed the decoupling of tensile and shear properties, showing an increase in both moduli after UVX in human donor corneas.^42^ In addition, the model quantified lower values of in-plane modulus when using a single-layer model than when using a two-layer model,^42^ therefore single-layer models may underestimate YM. The layer-dependent elasticity values (penetration depth of a crosslinker into the tissue) combined with the measurement method and the estimation model may explain the reported lack of efficacy of RGX in previous studies.

Some studies have shown RGX to be effective in enhancing overall corneal strength. Specifically, rabbit corneal stiffness increased in the range of 2.7 to 6.3-fold using air-puff deformation measurements with a 2-layer model,^23^ and in the range of 1.7 to 4.4-fold^20^^,^^21^^,^^22^ using tensile testing. Whether the findings in the cornea suggesting a great effect of corneal stiffness with cross-linking can be extrapolated to the sclera is unknown. However, we can determine the effectiveness of RGX in deep scleral tissue using OCE. By applying the multilayer wave propagation model^41^^,^^42^ to quantify the shear modulus from OCE measurements (e.g. using air-coupled ultrasonic OCE; Villegas L, et al. J of Vision 2023;23(11): ARVO E-Abstract 72), the stiffness of treated layers may be specifically determined. Future research will evaluate depth-resolved elastic changes in the SXL-treated sclera.

Although a significant increase in scleral stiffness has been shown after RGX, the cross-linked area may be shallow. Studies in the cornea have reported low penetration of Rose Bengal into the tissue (<130 µm)^20^^,^^21^^,^^43^ with the location of RGX-induced cell death being limited to the anterior stromal zone in rabbits (approximately 150 µm).^27^^,^^28^ However, Germann et al.^25^ found that collagen organization in corneal tissue after RGX extended deeper into the stroma (about 200 µm depth), that is, beyond the anterior region presumably cross-linked. Although the cross-linking mechanisms are still not well understood, the possible effective deeper cross-linking could be explained by an alternative oxidative mechanism (electron transfer process from amino acid in the collagen chains) within the collagen matrix suggested by molecular dynamics simulation.^44^ Deep structural changes in the sclera due to Rose Bengal have not been investigated. Therefore, our future work will include the use of fluorescence sectioning microscopy measurements to determine the penetration of Rose Bengal in the sclera.

In the current study, we chose to cross-link an area in the nasal or temporal region of the sclera and use the contralateral side as a control. We did not find statistical differences in the untreated nasal and temporal regions, unlike a previous report using OCE in porcine sclera.^45^ We did not find significant differences in the effect of cross-linking on either the temporal or nasal sclera, with only a tendency for the RGX-treated temporal sclera to show a slightly greater increase in stiffness (see Supplementary Table B1). However, regional effects were observed in the variation of swelling properties, where the lowest swelling rate (3.3 ± 0.4 %/min) with a reduction of approximately 4.6% per MPa was measured in the RGX-treated temporal scleral tissue. Previous reports of higher shear modulus change in temporal porcine sclera after SXL^45^ and higher collagen organization in rabbit temporal sclera than in the nasal region after cross-linking (Germann J, et al. IOVS 2021;62(8): ARVO E-Abstract 3276), suggest a relationship between collagen arrangement and out-of-plane modulus, which may be more affected by collagen organization than the in-plane modulus. Furthermore, the regional differences in saline uptake (lower in the temporal than nasal sclera after RGX) may be a result of structural differences (more cross-linked or interwoven collagen) in the temporal sclera after cross-linking.

The hydration process of the scleral tissue was affected by RGX and UVX only during the initial swelling phase. In a 40-minute rehydration period, the swelling ratio decreased by 12% after RGX and UVX. In a second slower swelling phase, the swelling ratio decreased by 9% after RGX and 6% after UVX. This two-phase hydration process (complementary to the two-phase drying model^46^) has been previously described in porcine corneas, where an initial nonsignificant decrease of 5% was observed, followed by a final increase in hydration of 10% after UVX.^47^ Thus, the swelling behavior found in this study could be explained by the rehydration process of collagen in which there appears to be an initial change in the interior of the fibrils and then a global change in collagen fibril length.^46^^,^^48^

Overall, photo-crosslinking with RGX or UVX resulted in approximately similar increases in stiffness. Although the highest stiffness values were quantified after UVX cross-linking, scleral stiffness after RGX/UVX varied by less than 1.7 MPa under physiological hydration conditions and with longer rehydration times. Surprisingly, scleral stiffness increased 5.9 MPa more after UVX than after RGX at shorter rehydration times. In contrast, a similar swelling rate (3.4 ± 0.5%/min vs. 3.5 ± 0.4%/min) and ratio (142.2 ± 20.8% vs. 152.3 ± 20.2%) were observed in the RGX-treated and UVX-treated tissue, respectively. These results suggest that the influence of UVX on scleral stiffness may be related to the tissue dehydration/rehydration process itself. Hayes et al.^47^ hypothesized that UVX cross-linking occurs only at the fibril surface and within the proteoglycan coating (outer limit of the coating around 36–80 nm^46^). Therefore, tighter molecular packing around the fibrils (due to UVX) would reduce the amount of water introduced into the interfibrillar space (during rehydration), limit collagen fibril movement, and improve overall tissue strength at the onset of the rehydration period. An additional confounding factor with UVX is the reduced tissue hydration (well-studied in the cornea) in the presence of dextran due to its hyperosmolarity, which is likely to affect swelling, particularly shortly after treatment.

A limitation of this study is that a tensile test is a destructive technique with a direct influence on hydration^29^^,^^31^ and mechanical properties.^49^^,^^50^ Specifically, a previous study concluded that uniaxial tensile test estimated lower scleral stiffness than the inflation test.^49^ Whereas the intact eye in inflation tests more closely approximates physiological conditions, the scleral properties reconstructed from inflation tests are necessarily influenced by assumptions regarding the cornea^51^^,^^52^ and ocular tissues as well as the preconditioning response during inflation.^50^ Conversely, the tensile test provides us with a direct quantification of the YM of tissues that other techniques can only obtain through inverse viscoelastic reconstruction techniques from air-puff deformation^23^^,^^24^^,^^53^ or OCE measurements.^40^^,^^42^ In addition, some studies have identified a preferential orientation of scleral collagen fibers.^54^^,^^55^ The equatorial orientation (measured here) has been reported to be stiffer than the meridional orientation estimated in scleral tissue by biaxial mechanical testing^56^ and by OCE (Villegas L, et al.; unpublished data; 2023). Certainly, tensile tests should be complemented with new techniques which allow quantification of mechanical properties in several directions simultaneously and avoid destruction of the ocular tissue.

In summary, RGX induced significant scleral stiffness similar to that induced by UVX in a rabbit model. The influence of RGX and UVX on the elastic and swelling properties of nasal and temporal sclera was quantified by hydration and tensile tests. The improvement in mechanical properties of scleral tissues was comparable after natural hydration and after prolonged hydration. Rose Bengal and green light irradiation may represent a potential therapeutic approach to delay the progression of myopia, but further research is needed to optimize the application of the treatment and to evaluate its biochemical effects on the retina and surrounding ocular tissues.

## Supplementary Material

Supplement 1
