# Supplementary material for: Effects of Different Scleral Photo-Crosslinking Modalities on Scleral Stiffness and Hydration
Source: Invest Ophthalmol Vis Sci. 2024 Jul 3;65(8):8. doi: 10.1167/iovs.65.8.8 (PMC11223619; doi:10.1167/iovs.65.8.8)
Supplement: Supplement 1 [file iovs-65-8-8_s001.pdf]

Effects of Different Scleral Photo-Crosslinking Modalities on Scleral Stiffness and Hydration

Lupe Villegas [1,3], James A. Germann[1], Susana Marcos[1,2]
1 Instituto de Óptica, Consejo Superior de Investigaciones Científicas, Madrid, Spain
2 The Center for Visual Science; The Institute of Optics; Flaum Eye Institute. University of Rochester, NY, United States
3 Universidad Politécnica de Madrid, Madrid, Spain

SUPPLEMENTARY MATERIAL

A. Estimated of Strain

To compare Young's modulus measured from rabbit scleral strips to intact eyes under an intraocular pressure (IOP) of 15mmHg, the relationships between IOP and stress-strain was determined by approximation. The shape of the sclera can be modeled as a spherical shell of radius r and thickness t filled with an aqueous liquid. The tension σ over the sclera due to the internal surface pressure P (in this case IOP) can be estimated using the Young–Laplace equation: $\sigma = \frac{P\,r}{2t}$. Considering the same applied external force $\sigma = A(e^{B\varepsilon} - 1)$ and joining the equations, $\frac{P\,r}{2t} \approx AB\varepsilon(1 + B\varepsilon)$. The radius[1] $r = 8.12mm$ and the internal pressure[2] $15 \leq P \leq 25\,mmHg$ are taken from the literature, while the parameters t, A, and B are taken from our calculated stress-strain curves for naïve scleral tissue of rabbit eyes ($t = 315 \pm 48\,\mu m$ and $A = 0.0016 \pm 0.0012\,MPa,\;\; B = 59.6 \pm 10.9$, see Section C). Strain was estimated at $6\% \leq \varepsilon \leq 8\%$. We calculated strains around 8% for comparison.

B. Tables of Young's Modulus Values

		**Photo-crosslinking treatment**		
		**RGX**	**UVX**	**Virgin**
**Zone**	**Region**	**Young's Modulus at 8% of strain (MPa)**	**Young's Modulus at 8% of strain (MPa)**	**Young's Modulus at 8% of strain (MPa)**
**Untreated sclera**	**Nasal**	3.2(1.5-4.9)	7.9(3.9-11.8)	5.8(2.9- 8.8)
	**Temporal**	4.9(2.2-7.6)	7.9(4.5-11.3)	6.1(2.2-10.0)
	Total	**4.1 (2.7-5.4) †**	**7.9(5.5-10.3)†**	**6.0(3.8-8.1)**
**Treated sclera**	**Nasal**	9.3(4.9-13.8)	14.8(11.8-17.8)	
	**Temporal**	9.6(4.3-14.9)	15.2(11.8-18.6)	
	Total	**9.5(7.3-11.7)†**	**15.0(11.9-18.2)†**	

Table B.1. Mean values (and 95% confidence intervals) of Young's modulus for untreated and treated scleral tissue in nasal and temporal regions. Multiple comparisons in zones revealed statistically significant differences in columns between labeled values (†: p≤.013).

		Young's Modulus at 8% of strain (MPa)			
		RGX		UVX	
Zone	Region	after 40 min rehydration	after 100 min rehydration	after 40 min rehydration	after 100 min rehydration
Untreated sclera	Nasal	7.6(2.8-12.4)	6.3(3.3-9.2)	8.2(1.1-15.4)	6.6(4.5-8.7)
	Temporal	8.7(4.2-13.2)	5.2(4.4-6.0)	5.2(0.03-10.5)	5.9(3.1-8.6)
	Total	**8.1(5.6-10.7) †**	**5.7(4.5-7.0)†**	**6.7(3.4-10.1)†**	**6.2(5.0-7.5)†**
Treated sclera	Nasal	14.0(5.8-22.1)	13.7(9.8-17.6)	19.7(11.9-27.5)	11.4(5.7-17.8.6)
	Temporal	13.9(7.3-20.4)	10.8(8.3-13.4)	17.0(6.0-28.0)	11.8(3.1-8.6)
	Total	**13.9(9.9-17.9)†**	**12.3(10.2-14.4)†**	**18.4(13.6-23.2)†**	**11.6(9.5-13.7)†**

Table B.2. Mean values (and 95% confidence intervals) of Young's modulus for untreated and treated scleral tissue in nasal and temporal regions. Multiple comparisons in zones revealed statistically significant differences in columns between labeled values (†: p≤.004).

C. Uniaxial Tensile Test of Virgin Scleral Tissue

Six enucleated rabbit eyes from adult New Zealand white rabbits (2-3 kg) were used within 24h post-mortem. Muscles and conjunctival tissue were removed from the sclera before the measurements, and regions were identified: temporal and nasal. Strips were extracted from posterior sclera (see Section 2.1) and subjected to uniaxial tensile test. Fig C.1. shows that there is no significant (p=0.93) difference in stiffness. Young's modulus at 8% strain was 5.8±2.8 MPa in nasal region and 6.1±3.7 MPa in temporal region (see Fig. C.1b).

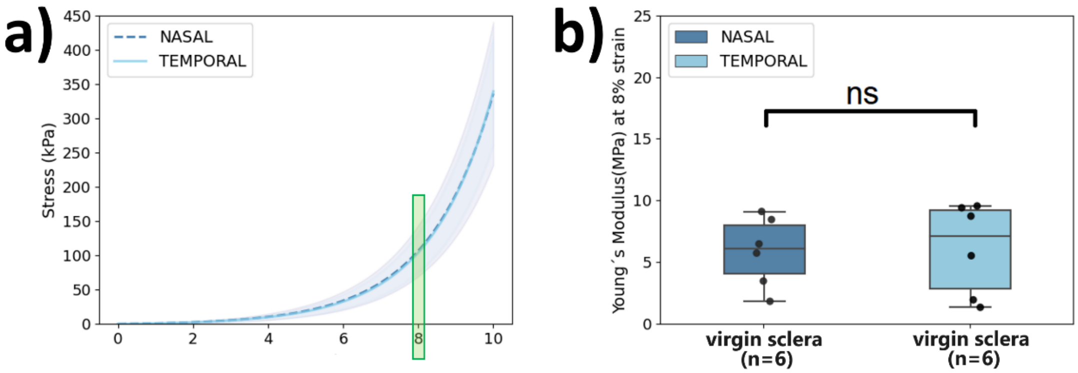

Fig C.1. No nasal/temporal differences in stiffness in virgin scleral tissue. (a) Uniaxial stress-strain curves of scleral strips from naïve tissues. Scleral strips were obtained from the posterior nasal (dark-blue dashed line) and temporal (light-blue solid line) regions. Young's modulus was calculated from the slope of the stress-strain curves at 8% strain (represented as a green line). **(b)** Here are shown the boxplots of estimated Young's modulus for nasal (dark-blue, n=6 eyes) and temporal (light-blue, n=6 eyes). The line inside the box is the median, and data points are shown as black circles. Each data point represents the mean modulus of two strips in each region per eye. A repeated

measures ANOVA determined that mean Young's modulus did not have a statistically significant difference between eyes ($F(1, 5) = 0.049$, $p = 0.833$). "ns": means not significant.

D. Table of Swelling Rate Values

		Photo-crosslinking treatment	
		RGX	UVX
Zone	Region	Swelling rate (%/min)	Swelling rate (%/min)
Untreated sclera	Nasal	4.0(3.6-4.5)	4.1(3.8-4.5)
	Temporal	3.7(3.1-4.2)	3.9(3.5-4.4)
	Total	**3.8(3.5-4.2) †**	**4.0(3.8-4.3)†**
Treated sclera	Nasal	3.4(3.0-3.8)	3.6(3.3-3.9)
	Temporal	3.3(3.1-3.6)	3.5(3.2-3.8)
	Total	**3.4(3.1-3.6)†**	**3.5(3.3-3.7)†**

Table D.1. Mean values (and 95% confidence intervals) of swelling rate for untreated and treated scleral tissue in nasal and temporal regions. Multiple comparisons in zones revealed statistically significant differences in columns between labeled values (†: $p \leq .003$).

E. Thickness, width and length data of strips

The thickness was measured at the center of each strip immediately before the tensile test. Thickness was measured (three times) with a micrometer (digital, Mitutoyo, model 293-240-30). Mean values (coefficient of variation <3%) were used to quantify the cross-sectional area (thickness × width of the center of the scleral strip). During the hydration-tensile test, the weight of the strip was repeatedly checked twice between thickness measurements to be sure of the hydration value of the strip before it was mounted in the tensile test.

In tensile test, thickness values were not statistically significantly different in RGX-treated (337.4±31.3 µm vs. 341.2±28.5 µm, *p=.43*) and UVX-treated (300.3±29.5 µm vs. 324.5±37.4 µm, *p=.23*) sclera compared to untreated sclera. There were no statistically significant differences in thickness between temporal and nasal regions in virgin sclera (324.2±55.0 µm and 306.8±44.0 µm, *p=.58*), see Table E1.

In hydration-tensile test, there was no statistically significant difference in thickness between RGX-treated (281.0±19.6 µm vs. 290.6±36.1 µm, *p=.76*) and UVX-treated (280.6±15.2 µm vs. 280.9±24.1 µm, *p=.27*(UVX-40min)) sclera compared to untreated sclera after 40 minutes of rehydration, nor after 100 minutes of rehydration in RGX-treated (298.1±12.7 µm vs. 294.2±23.6 µm, *p=.21*) and UVX-treated sclera (307.9±22.3 µm vs. 321.9±22.0 µm, *p=.29*) sclera compared to untreated sclera, see Table E2.

		Photo-crosslinking treatment		
		RGX	UVX	Virgin
Zone	Region	Thickness (µm)	Thickness (µm)	Thickness (µm)
Untreated sclera	Nasal	343(317-369)	323(259-387)	324(266-382)
	Temporal	339(303-375)	326(298-354)	307(261-353)
	Total	**341(323-359)**	**324(298-351)**	**315(285-346)**
Treated sclera	Nasal	323(288-358)	294(264-324)	
	Temporal	352(327-376)	307(262-351)	
	Total	**337(317-357)**	**300(279-321)**	

Table E.1. Mean values (and 95% confidence intervals) of thickness for untreated and treated scleral tissue in nasal and temporal regions. Comparisons revealed no statistically significant main effect among the thickness (repeated measures ANOVA, *p=.43* (RGX), *p=.23*(UVX), and one-way ANOVA *p=.58*(Virgin)).

		Thickness (µm)			
		RGX		UVX	
Zone	Region	after 40 min rehydration	after 100 min rehydration	after 40 min rehydration	after 100 min rehydration
Untreated sclera	Nasal	289(236-342)	283(256-310)	268(221-316)	333(329-338)
	Temporal	292(251-334)	305(278-332)	293(284-303)	310(266-354)
	Total	**290(265-317)**	**294(277-311)**	**281(261-301)**	**322(303-340)**
Treated sclera	Nasal	284(258-310)	297(279-315)	285(264-305)	303(253-354)
	Temporal	278(253-303)	299(285-314)	277(248-305)	313(296-328)
	Total	**281(267-295)**	**298(289-307)**	**281(268-293)**	**308(289-326)**

Table E.2. Means (and 95% confidence intervals) of thickness for untreated and treated scleral tissue in nasal and temporal regions. Comparisons revealed no statistically significant main effect among the thickness (repeated measures ANOVA, *p=.76*(RGX-40min), *p=.21*(RGX-100min), *p=.27*(UVX-40min), *p=.29*(UVX-100min)).

F. Tensile testing in combination with hydration process for scleral strips

After photo-crosslinking treatment, scleral strips were extracted from treated and untreated regions of the eyes, see Figure. F1a and F1b. First, the samples were dehydrated (for 24 hours at 21.0 ± 2.0^{o}C and 37.2 ± 1.1% humidity), see dehydrated scleral samples in Fig. F1c. The strips were dried on a flat surface in a petri dish, which in almost all cases accurately maintained the flatness of the sample for easy thickness measurement. Each scleral strip was then rehydrated by instillation of 10 µL of phosphate-buffered saline (Fig. F1d left) and then weighed every 10 minutes. After 40 or 100 minutes of rehydration (Fig. 1d right), each strip was mounted for uniaxial tensile testing (Fig. F1e).

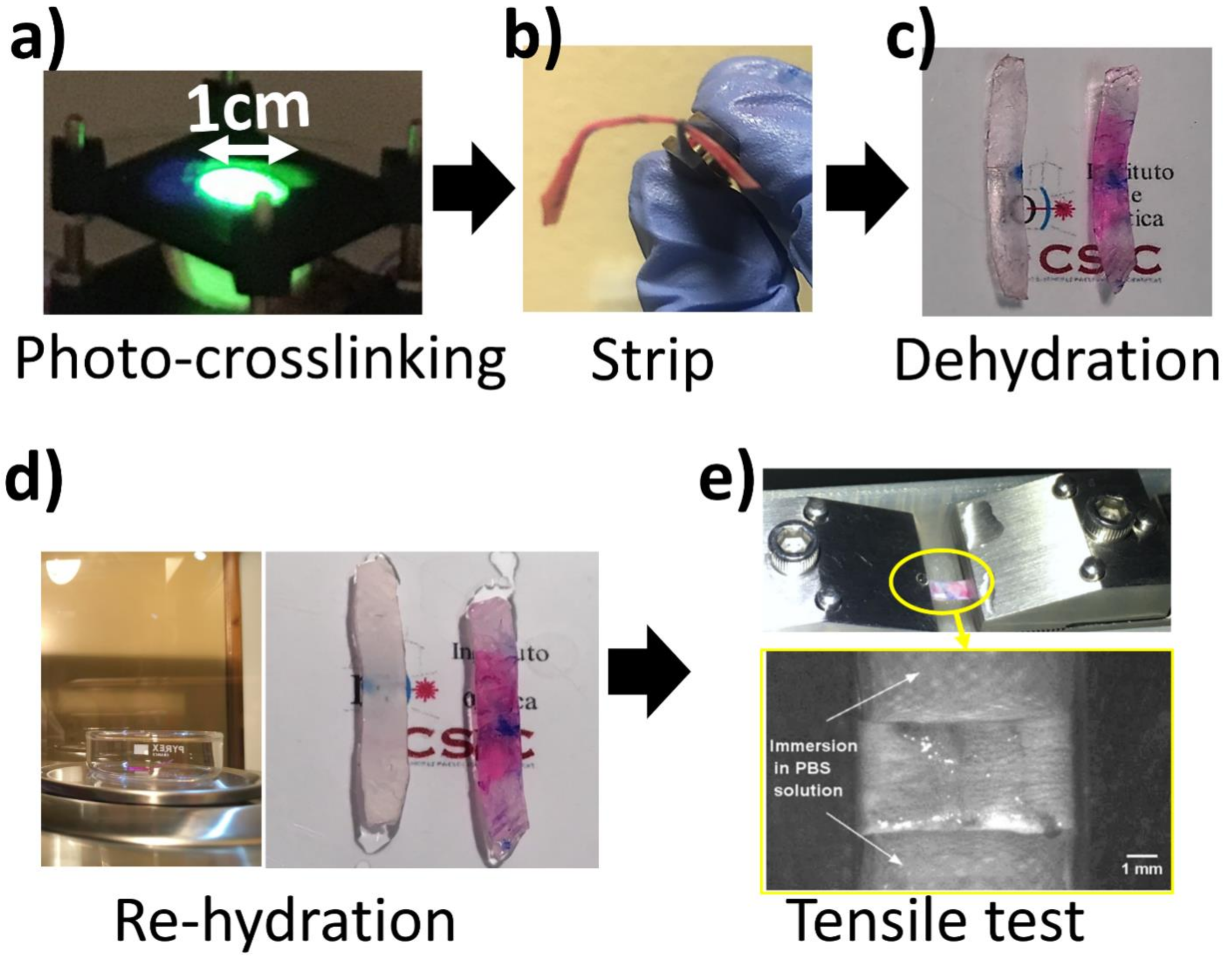

Fig. F.1 Schematic sequence of hydration-tensile test. (a) Rabbit globe eyes were treated by photo-crosslinking. **(b)** Strips of untreated and treated scleral tissue were dissected from nasal and temporal regions of the rabbit eyes. **(c)** Strips were dehydrated (24 h), and then **(d)** rehydrated for 40 min or 100 min. Finally, strips were mounted (**e)** in a uniaxial stretcher.

Supplementary References

1. Barathi A, Thu MK, Beuerman RW. Dimensional Growth of the Rabbit Eye. Cells Tissues Organs. 2002;171(4):276-285.
2. Vareilles P, Conquet P, Le Douarec JC. A method for the routine intraocular pressure (IOP) measurement in the rabbit: Range of IOP variations in this species. Exp Eye Res. 1977;24(4). doi:10.1016/0014-4835(77)90149-X
